# Cytokine Pattern of T Lymphocytes in Acute *Schistosomiasis mansoni* Patients following Treated Praziquantel Therapy

**DOI:** 10.1155/2013/909134

**Published:** 2013-01-20

**Authors:** Denise Silveira-Lemos, Matheus Fernandes Costa-Silva, Amanda Cardoso de Oliveira Silveira, Mauricio Azevedo Batista, Lúcia Alves Oliveira-Fraga, Alda Maria Soares Silveira, Maria Carolina Barbosa Alvarez, Olindo Assis Martins-Filho, Giovanni Gazzinelli, Rodrigo Corrêa-Oliveira, Andréa Teixeira-Carvalho

**Affiliations:** ^1^Laboratório de Biomarcadores de Diagnóstico e Monitoração, Centro de Pesquisas René Rachou, FIOCRUZ, Barro Preto, 30190-002 Belo Horizonte, MG, Brazil; ^2^Laboratório de Imunologia Celular e Molecular, Centro de Pesquisas René Rachou, FIOCRUZ, 30190-002 Belo Horizonte, MG, Brazil; ^3^Laboratório de Imunologia e Genômica de Parasitos, Departamento de Parasitologia, Instituto de Ciências Biológicas (ICB), Universidade Federal de Minas Gerais, Belo Horizonte, MG, Brazil; ^4^Laboratório de Imunoparasitologia, Departamento de Ciências Biológicas, NUPEB, Instituto de Ciências Exatas e Biológicas, Universidade Federal de Ouro Preto, Ouro Preto, MG, Brazil; ^5^Núcleo de Pesquisa em Imunologia, Faculdade de Ciências da Saúde, Universidade Vale do Rio Doce, Governador Valadares, MG, Brazil; ^6^Santa Casa de Misericórdia de Belo Horizonte, Belo Horizonte, MG, Brazil

## Abstract

Acute schistosomiasis is associated with a primary exposure and is more commonly seen in nonimmune individuals traveling through endemic regions. In this study, we have focused on the cytokine profile of T lymphocytes evaluated in circulating leukocytes of acute *Schistosomiasis mansoni*-infected patients (ACT group) before and after praziquantel treatment (ACT-TR group). Our data demonstrated increased values of total leukocytes, eosinophils, and monocytes in both groups. Interestingly, we have observed that patients treated with praziquantel showed increased values of lymphocytes as compared with noninfected group (NI) or ACT groups. Furthermore, a decrease of neutrophils in ACT-TR was observed when compared to ACT group. Analyses of short-term *in vitro* whole blood stimulation demonstrated that, regardless of the presence of soluble *Schistosoma mansoni* eggs antigen (SEA), increased synthesis of IFN-**γ** and IL-4 by T-cells was observed in the ACT group. Analyses of cytokine profile in CD8 T cells demonstrated higher percentage of IFN-**γ** and IL-4 cells in both ACT and ACT-TR groups apart from increased percentage of IL-10 cells only in the ACT group. This study is the first one to point out the relevance of CD8 T lymphocytes in the immune response induced during the acute phase of schistosomiasis.

## 1. Introduction

Schistosomiasis is a tropical parasitic chronic disease, caused by worms of the genus *Schistosoma*. The infection is endemic in tropics and subtropics around the world and is present in Africa, South America, Arabia, and Asia. There are approximately 200 million people infected worldwide, and the number of deaths caused by schistosomiasis is estimated at around 41.000 annually [1]. *Schistosoma mansoni* worms dwell in peri-intestinal venules and cause intestinal, hepatointestinal, and hepatosplenic schistosomiasis [2].

Brazil is the country most affected by schistosomiasis in the Americas, with 1.5 million people infected and more than 36 million at risk of acquiring infection [3, 4]. In the state of Minas Gerais, *Schistosomiasis mansoni* is prevalent in 519 municipalities, with an estimated number of one million people infected [5].

The propagation and maintenance of schistosomiasis in a given region are conditional of many factors such as appropriate climate, socioeconomic conditions, rapid spread of intermediate hosts, sanitary conditions, and water supply [6]. Tourist activity in endemic areas may be an important contributing factor to the propagation of outbreaks/cases of schistosomiasis [7–10]. 

In endemic areas for schistosomiasis, people develop the chronic phase of infection (which begins around six months after exposure) and have different clinical manifestations. People who had never had previous contact with the parasite develop acute schistosomiasis when contracting the infection [11, 12]. 

The acute phase starts after cercarial infection, and local urticaria may appear in a few hours. Between one and four weeks after infection, the migrating and maturing schistosomula can cause a systemic hypersensitivity reaction with fever, general weakness, headache, nausea, vomiting, diarrhea, and dry cough [2, 9]. In this phase, individuals present a mixed cytokine profile with predominance of Th1 type of CD4^+^ T-cell differentiation [13, 14]. 

In the context of cytokine milieu triggered by *S. mansoni* infection, it has been suggested that interleukin (IL)-4 upregulates fibroblast chemokine, matrix protein expression, and collagen, which implies that IL-4 is a crucial cytokine for granuloma formation [15] and reduces the cellular proliferative response to soluble egg antigens (SEA) [13]. IL-5 in schistosomiasis induces liver fibrosis [16]. IL-10 has been shown to be a major cytokine during infection with downregulatory activity of both Th1 and Th2 T cell subpopulations [17]. Interferon gamma (IFN-*γ*) is related to the activation of macrophages and plays a key role in the protective mechanism against periportal fibrosis, whereas the proinflammatory tumor necrosis factor-alpha (TNF-*α*) may aggravate the disease [18].

As regards the cytokine profile after specific chemotherapy, it has been suggested that peripheral blood mononuclear cells (PBMC) from patients with acute infection responded to SEA and soluble worm antigen preparation (SWAP) by producing significantly higher amounts of IFN-*γ* and IL-10. However, IL-5 was detected only in SEA-stimulated cultures, and little or no IL-4 was detected in SEA or SWAP-stimulated cells [19]. De Jesus et al. [9] suggested that most patients after specific chemotherapy for schistosomiasis spontaneously released high levels of TNF-*α*, IL-1, and IL-6. In addition, detectable levels of IFN-*γ* were present in the supernatants of unstimulated PBMC from these patients. Stimulation of PBMC from patients with acute disease only induced higher levels of IFN-*γ* upon SEA stimulation [9]. 

In the present study, we evaluated the cytokine profile (IFN-*γ*, TNF-*α*, IL-4, IL-5, and IL-10) of T lymphocytes and their subsets as well as the ultrasonographic features of patients with acute schistosomiasis infection before and after praziquantel treatment. 

## 2. Population, Material, and Methods

### 2.1. Study Population

The patients evaluated in this study were infected accidentally by *S. mansoni* in the country village of São Geraldo da Piedade, an endemic area for schistosomiasis, located in the east of the state of Minas Gerais (MG), Brazil, during the traveling in a holiday. This people reported swimming in a stream that probably harbors cercariae released by infected snails. In this area 60% of *Biomphalaria glabrata* are infected and the chronic disease is frequent in the population. These individuals went to the hospital because they felt very bad, with acute symptoms. The patients did not live in areas of active transmission of *S. mansoni*. All patients showed clinical symptoms associated with early *S. mansoni* infection such as fever, diarrhoea, headache, and nausea. Only patients who had positive results for quantitative parasitological stool examinations for *S. mansoni* by the Kato-Katz method [20] and signed an informed consent were included in this study. The group of acutely *S. mansoni*-infected patients (ACT) without praziquantel treatment consisted of twenty-one individuals, five women and sixteen men, with ages ranging from 13 to 44 years, with parasite load ranging from 12 to 1812 eggs per gram of feces (epg). After blood sample collection, all *S. mansoni*-infected individuals received specific therapeutic treatment with praziquantel in the standard Brazilian dose (50–60 mg/Kg praziquantel), regardless of whether or not they were going to participate in this study [21]. One month after praziquantel treatment, blood was collected from a group of patients (ACT-TR) composed of three women and four men, aged between 12 and 40 years. An additional noninfected group (NI) composed of nineteen individuals, eleven woman and eight men, with age ranging from 19 to 45 years, who were healthy blood donors contacted at the Hemominas Blood Bank Foundation in Belo Horizonte, MG, Brazil, participated in the study. All noninfected volunteers were included after three consecutive negative parasitological exams for *S. mansoni* infection apart from negative serology for Chagas disease, leishmaniasis, human immunodeficiency virus infection, and hepatitis. This study was approved by the Ethics Committees at the Oswaldo Cruz Foundation (FIOCRUZ), the School of Medicine at the Federal University of Minas Gerais (UFMG), and the Brazilian National Committee on Ethics in Research (CONEP).

### 2.2. Ultrasonographic Analysis

Ultrasonographic evaluation was performed in all individuals using a Nemio SSA/550A ultrasound machine (Toshiba) with a 3 MHz sector probe. Liver size, portal vein diameter, thickness of central walls and peripheral portal branches, spleen size, and splenic vein diameter were assessed as described in previous studies [22, 23]. Liver span was measured both in the midclavicular line and the midline. The liver was also examined for surface smoothness. Portal vein diameter was measured at its entrance into the liver and its bifurcation to the liver. Oblique and longitudinal scans of the left upper quadrant were used to evaluate the spleen. The gallbladder was examined for wall thickness and stones. Periportal thickness was evaluated according to established criteria [22–24]. All individuals were examined by the same physician.

### 2.3. Evaluation of Hematological Parameters

 Hemograms were performed with an automated blood cell counter (Coulter MD18, USA), using whole blood collected in 5 mL vacutainer tubes containing the anticoagulant ethylenediamine tetraacetic acid (EDTA) (Becton Dickinson Biosciences, San Diego, CA, USA). The parameters measured were total leukocytes counts and differential analysis of leukocyte subsets including the absolute counts of eosinophils, neutrophils, lymphocytes, and monocytes.

### 2.4. Preparation of Antigens

*S. mansoni* eggs were isolated from the livers of infected mice, exposed 8 weeks previously to cercariae, homogenized, and ground in cold phosphate-buffered saline (PBS). The clear supernatant fluid resulting from high-speed centrifugation of the homogenate at 50.000 ×g for 1 h at room temperature was named soluble egg antigens (SEA) and stored at −70°C until use [25].

### 2.5. Monoclonal Antibodies

 The experiments used monoclonal antibodies (mAbs) against both human cell surface markers, including CD3 (UCTH-1), CD4 (SK3), and CD8 (SK1) labeled with fluorescein isothiocyanate (FITC), and intracytoplasmic cytokines including IL-4 (MP4-25D2), IL-5 (TRFK5), IL-10 (JES3-9D7), IFN-*γ* (B27), and TNF-*α* (Mab11) labeled with phycoerythrin (PE). Isotypic controls were also used in all experiments, including mouse IgG1 (679.1Mc7) and IgG2a (UCTH-1) labeled with FITC and PE, respectively. All mAbs and controls were purchased from Becton Dickinson Biosciences Pharmingen (San Diego, CA, USA).

### 2.6. Short-Term Whole Blood Culture *In Vitro* and Intracellular Cytokine Immunostaining

Short-term cultures *in vitro* and cytokine immunostaining were performed as described by Silveira-Lemos et al. [26]. Briefly, five hundred microliters of peripheral blood samples collected into Vacutainer tubes containing heparin sodium salt (Becton Dickinson, Mountain View, CA, USA) were dispensed into individual 17 × 100 mm polypropylene tubes (Falcon 2059 - Becton Dickinson, Mountain View, CA, USA). Short-term cultures were performed in the absence (control cultures) or in the presence of *S. mansoni* eggs derived antigens (SEA). For this purposes, 500 *μ*L of whole blood samples were incubated in the presence of 500 *μ*L of RPMI 1640 plus Brefeldin A, BFA (Sigma Chemical Company, St Louis, MO, USA) at a final concentration of 10 *μ*g/mL. The blood samples were incubated for 4 h at 37°C in a 5% CO_2_ humidified incubator. These conditions were chosen considering that the detection of cytokines, particularly in the absence of exogenous stimuli, may reflect the events of cytokine production by blood leukocytes *in vivo*. Antigen-specific stimulation was performed by previous *in vitro* incubation of 500 *μ*L whole blood aliquots in the presence of SEA at a final concentration of 25 *μ*g/mL for 1 h at 37°C in a 5% CO_2_ humidified incubator. Following the addition of BFA (Sigma Chemical Company, St Louis, MO, USA) at a final concentration of 10 *μ*g/mL, blood samples were incubated for an additional 4 h at 37°C in a 5% CO_2_ humidified incubator. Prior to immunostaining for intracellular cytokine, all cultures, including control and SEA stimulated, were treated with 2 mM EDTA (Sigma Chemical Company, St. Louis, MO, USA) and kept at room temperature for 15 min, to stop cell activation process.

Following the short-term *in vitro* stimulation, cultured whole blood samples were washed with 6 mL of FACS buffer containing PBS supplemented with 0.5% Bovine Serum Albumin (BSA) and 0.1% sodium azide (Sigma Chemical Company, St. Louis, MO, USA), by centrifugation at 600 ×g at room temperature for 7 min. After resuspension in 1 mL of FACS buffer, 400 *μ*L aliquots were dispensed into one 12 × 75 mm polystyrene tube (Becton Dickinson, Mountain View, CA, USA) and labeled in the dark for 30 min at room temperature with the manufacture's recommended amount of mAbs anti-CD3, anti-CD4, and anti-CD8-FITC. Following the erythrocyte lyses step, the cells were incubated with 3 mL of FACS permeabilizing solution, containing FACS buffer supplemented with 0.5% of saponin (Sigma Chemical Company, St. Louis, MO, USA) in the dark for 10 min at room temperature. Following incubation, the samples were centrifuged at 600 ×g at room temperature for 7 min, the supernatant gently decanted and 3 mL of FACS buffer added to the resuspended pellet. After centrifugation, the pellet was resuspended and 30 *μ*L aliquots of cell suspension distributed in 96 wells U bottom microplate (Falcon-Becton Dickinson, Mountain View, CA, USA) and stained with 20 *μ*L of PE-labeled anticytokine mAb (anti-IL-4, anti-IL-5, anti-IL-10, anti-IFN-*γ*, and anti-TNF-*α*,). The cells were incubated in the dark for 30 min at room temperature. After incubation, the cells were washed with 150 *μ*L of FACS permeabilizing solution followed by 200 *μ*L of FACS buffer. After washing, the stained cells were fixed in 200 *μ*L of FACS fix solution and the samples stored at 4°C in the dark and analyzed by flow cytometry.

### 2.7. Data Collection and Analysis

Immunostained samples were run in a FACScan three-color detection flow cytometer (Becton Dickinson, San Jose, CA, USA). Data were collected and analyzed with CellQuest software (after collection of 50.000 events/sample). Cytokine-expressing T lymphocytes, including CD3^+^, CD4^+^, and CD8^+^ T cells, were identified by dual color immunophenotyping with FL1/FITC-labeled antihuman cell surface markers against CD3, CD4 and CD8 mAbs together with FL2/PE-labeled anticytokine mAbs. Lymphocyte gating was initially based on lymphocyte selection by forward scatter (FSC) versus side light scatter (SSC) properties on dot plot distributions, where they are confined into a region of low size and complexity. Further analysis of lymphocytes subpopulations was based on their selective staining with FITC-labeled anti-CD3, anti-CD4, and anti-CD8 mAbs. Cytokine-expressing cell subsets were quantified using FL1 cell surface markers versus FL2 anti cytokine-PE dot plots by setting quadrants to segregate FL2 positive and negative cells based on the negative control immunostaining. The results are expressed as the percentage of lymphocyte subsets expressing a given cytokine. 

### 2.8. Statistical Analysis

The statistical analysis was performed with software GraphPad PRISM 5.00 for Windows (La Jolla, CA, USA). Considering the parametric nature of the data, all results were analyzed by the ANOVA test followed by Tukey's multiple comparison test. Significance was defined at *P* < 0.05.

## 3. Results

### 3.1. Ultrasonographic Features of the Study Group

Table 1 shows the most important ultrasonographic features from ACT in comparison with control group. Our data demonstrated that ACT had significant increase in the measurement (in mm) of the longitudinal right lobe of liver (ACT: 147.4 ± 11.5; CT: 134.3 ± 10.5), anteroposterior left lobe of liver (ACT: 53.7 ± 7.6; CT: 42.7 ± 4.4), the anteroposterior right lobe of liver (ACT: 84.3 ± 9.6; CT: 70.3 ± 10.6), size of longitudinal and the anteroposterior spleen (ACT: 116.9 ± 15.6; CT: 88.1 ± 7.0 and ACT: 46.6 ± 8.5; CT: 33.7 ± 3.9, resp.), dimension of Hilar portal vein wall (ACT: 2.0 ± 0.1; CT: 1.5 ± 0.3, resp.) as compared with the CT group. No significant differences were found according to the sex categories.

### 3.2. Hematological Parameters during Acute *Schistosomiasis mansoni* Infection before and after Treatment with Praziquantel

**Figure 1:**
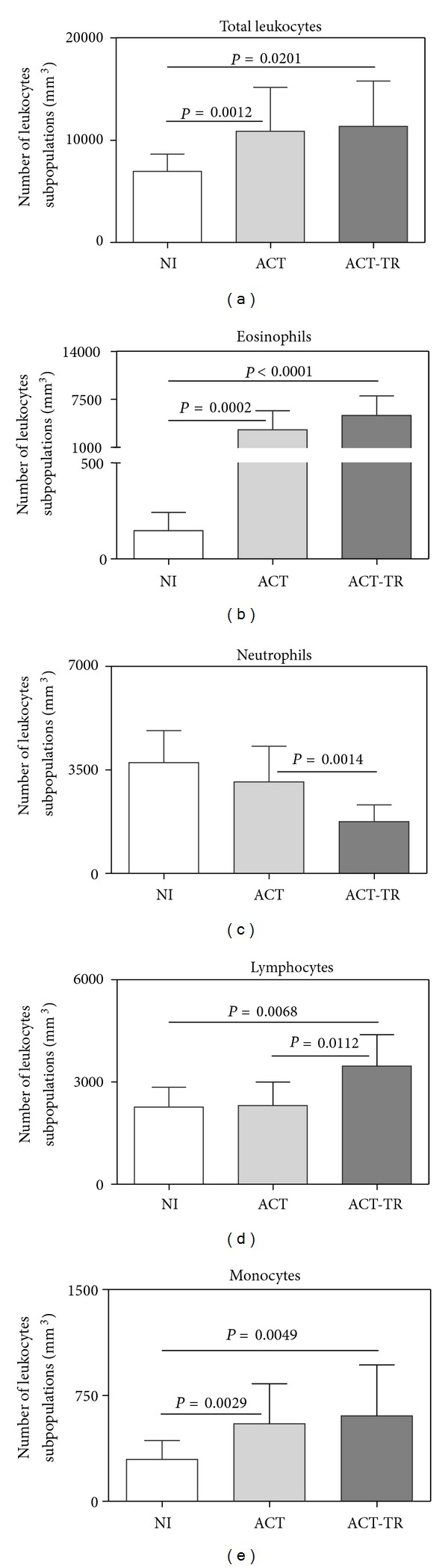
Analysis of hematological profile from patients with acute* Schistosomiasis mansoni *before (ACT, faint grey square = 21) and after praziquantel treatment (ACT-TR, grey square = 07) and noninfected individuals (NI, white square = 19). The results are shown in bar-plot format highlighting the mean counts of datasets/mm^3^ ± standard deviation. Statistically significant differences (connecting lines) observed between groups NI, ACT, and ACT-TR were considered at *P* < 0.05.

The results showed an increase in the values of total leukocytes from ACT and ACT-TR as compared with NI. A differential analysis of leukocyte subsets provides a more detailed description of the specific differences across cell types. The data showed increased counts of eosinophils and monocytes in the ACT and ACT-TR groups as compared with NI. Moreover, increased values were detected for lymphocytes from ACT-TR as compared with NI and ACT. Interestingly, after specific therapy, count of neutrophils in ACT-TR was decreased in comparison with NI (Figure 1). 

### 3.3. Cytokine Producing T Lymphocytes following *In Vitro* SEA Stimulation

Aiming to evaluate the impact of SEA stimulation in triggering proinflammatory and regulatory cytokines by T lymphocytes and the effect of the specific therapy for this profile, we have performed a detailed single cell flow cytometric analysis to quantify the frequency of cytokine^+^ cells for IFN-*γ*, TNF-*α*, IL-4, IL-5, and IL-10 after *in vitro* short-term incubation of whole blood samples focusing on T lymphocytes and their subsets (CD4^+^ or CD8^+^ T cells). 

**Figure 2:**
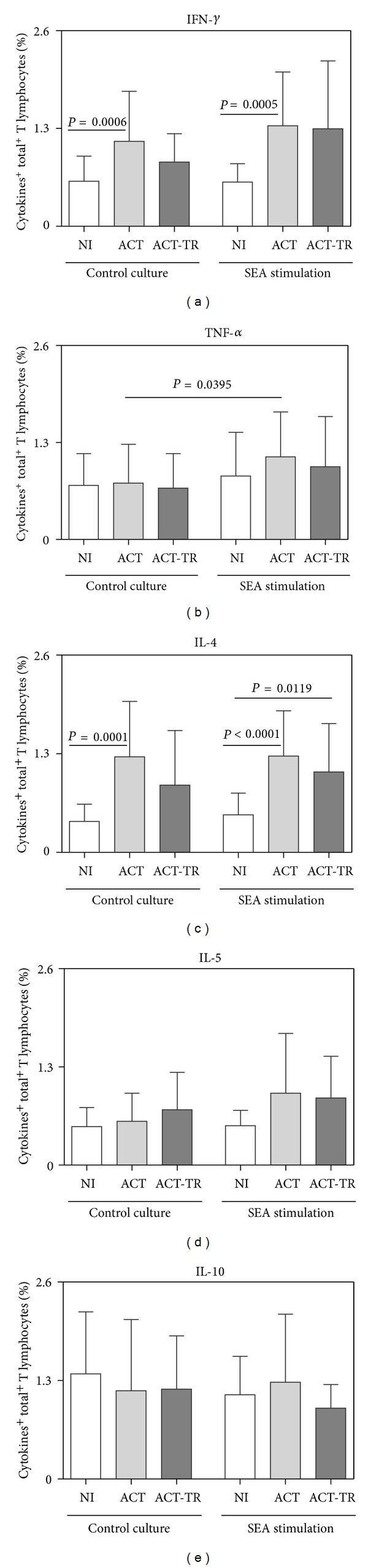
Cytokine pattern of circulating T lymphocytes from patients with acute *Schistosomiasis mansoni* before (ACT, faint grey square = 21) and after praziquantel treatment (ACT-TR, grey square = 07) and noninfected individuals (NI, white square = 19), following short-term *in vitro* cultivation in the absence (control culture) or presence of SEA stimulation. The results are shown in bar-plot format highlighting the mean percentage of datasets ± standard deviation. Statistically significant differences (connecting lines) observed between groups NI, ACT, and ACT-TR were considered at *P* < 0.05.

Regardless of the addition of SEA, the data revealed increased synthesis for IFN-*γ* and IL-4 by T cells in ACT as compared with NI (Figures 2(a) and 2(c), resp.). After SEA stimulation, we also observed increased synthesis of IL-4 in ACT-TR as compared with NI (Figure 2(c)). Moreover, the analysis of the impact of SEA addition to the cultures detected increased synthesis of TNF-*α* in ACT (Figure 2(b)). No significant differences were found according to the sex categories.

### 3.4. Cytokine Producing T CD4^+^ Lymphocytes following *In Vitro* SEA Stimulation

**Figure 3:**
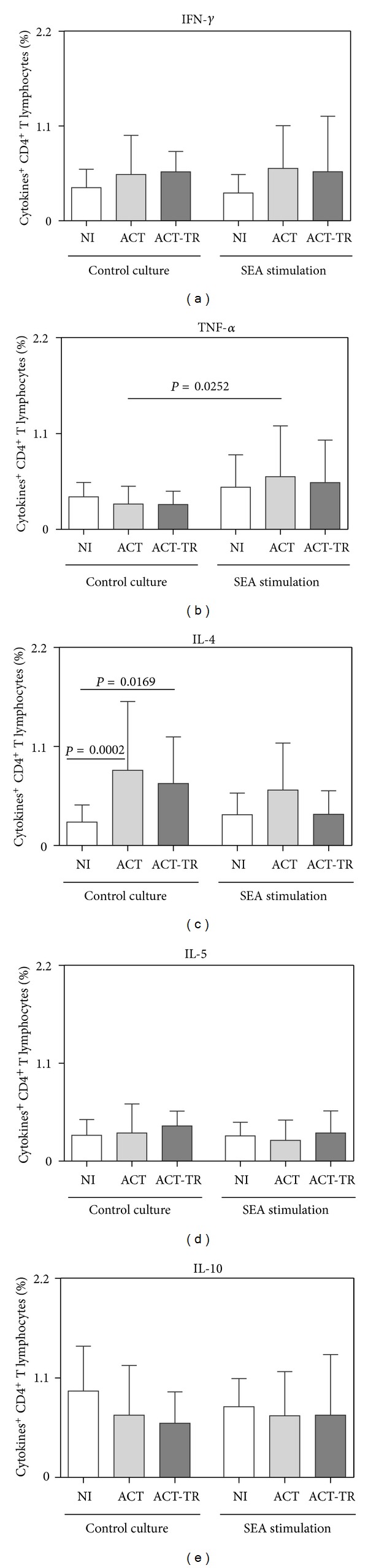
Cytokine pattern of circulating T CD4^+^-lymphocytes from patients with acute *Schistosomiasis mansoni* before (ACT, faint grey square = 21) and after praziquantel treatment (ACT-TR, grey square = 07) and noninfected individuals (NI, white square = 19), following short-term *in vitro* cultivation in the absence (control culture) or presence of SEA stimulation. The results are shown in bar-plot format highlighting the mean percentage of datasets ± standard deviation. Statistically significant differences (connecting lines) observed between groups NI, ACT, and ACT-TR were considered at *P* < 0.05.

The analysis of the results showed, in the control culture, increased synthesis of IL-4 by CD4^+^ T lymphocytes in ACT and ACT-TR groups as compared with NI (Figure 3(c)). Furthermore, after SEA stimulation, we observed increased synthesis of TNF-*α* by CD4^+^ T lymphocytes in ACT as compared with the control culture (Figure 3(b)). 

### 3.5. Cytokine Producing T CD8^+^ Lymphocytes following *In Vitro* SEA Stimulation

**Figure 4:**
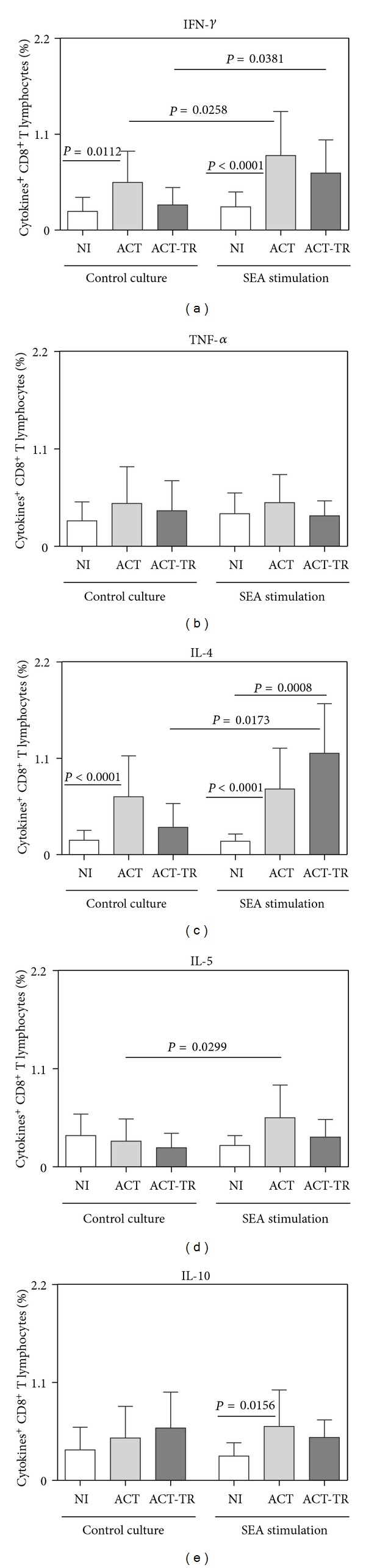
Cytokine pattern of circulating T CD8^+^-lymphocytes from patients with acute *Schistosomiasis mansoni* before (ACT, faint grey square = 21) and after praziquantel treatment (ACT-TR, grey square = 07) and noninfected individuals (NI, white square = 19), following short-term *in vitro* cultivation in the absence (control culture) or presence of SEA stimulation. The results are shown in bar-plot format highlighting the mean percentage of datasets ± standard deviation. Statistically significant differences (connecting lines) observed between groups NI, ACT, and ACT-TR were considered at *P* < 0.05.

The analysis of the cytokine profile in the CD8^+^T subset revealed a higher number of differences than in the CD4^+^ T cells. Regardless of SEA stimulation, increased synthesis of IFN-*γ* and IL-4 by CD8^+^ T lymphocytes was observed in ACT as compared with NI (Figures 4(a) and 4(c), resp.). Moreover, in cultures stimulated with SEA, increased synthesis of IL-4 was detected in ACT-TR as well as increased synthesis of IL-10 in ACT as compared with NI (Figures 4(c) and 4(e), resp.). After SEA stimulation, data analysis also showed increased synthesis of IFN-*γ* or IL-5 by CD8^+^ T lymphocytes as compared with the control culture (Figures 4(a) and 4(d), resp.). When the impact of SEA addition in the cultures from ACT-TR patient was investigated, increased synthesis of IFN-*γ* or IL-4 by CD8^+^ T lymphocytes were observed as compared with the control cultures (Figures 4(a) and 4(c), resp.).

## 4. Discussion

Acute schistosomiasis is a severe disease and the mechanism involved in its clinical manifestations is not completely understood. In the present study, we evaluated patients exposed to the same place of contaminated water, 40–60 days after exposure to cercaria. Different studies concerning this acute disease refer to groups of tourists, fishermen, or sailors originally from a nonendemic country who has visited a tropical zone [27–29]. However, as schistosomiasis is a focally distributed disease [29–31], the acute form is also diagnosed in inhabitants from endemic countries who do not live in endemic areas. The main clinical findings presented in our study such as headache, fever, diarrhea, and weight loss were consistent with those found by others authors [7, 9, 10, 29, 32].

Between two and eight weeks after a first contact with natural water infested by *Schistosoma* cercariae, susceptible infected patients may present a syndrome comprising of a period of 2 to 30 days of fever, diarrhea, toxemia and weakness, weight loss, abdominal pain, cough, myalgia, arthralgia, edema, urticaria, nausea/vomiting, and hepatosplenomegaly [33]. Thus, the diagnosis poses a challenge to physicians owing to the nonspecificity symptoms as well as the lack of positivity for *S. mansoni* eggs in feces from acute patients in the earlier stage of the infection. In this context, abdominal ultrasound imaging can be a complementary tool to assist the diagnosis of *S. mansoni* infection and its clinical monitoring. The ultrasonographic findings presented in this study were consistent with those described by Barata et al. [34] and Costa-Silva [32], which showed nonspecific increase in the size of liver and spleen (Table 1). Costa-Silva [32] showed that the ACT group had increased measurement of longitudinal left/right lobe of liver, size of longitudinal spleen as well as dimension of Hilar portal vein wall, and incipient periportal echogenic thickening, namely, grade I fibrosis. Thus, the data presented here suggest that alterations identified by analyses of ultrasonographic features, although not specific, are compatible with the acute phase of *Schistosomiasis mansoni* and provide a reliable complementary tool for the diagnosis and clinical followup of the disease.

Analyses of hematological parameters showed that ACT and ACT-TR groups presented increased values for total leukocytes, eosinophils, or monocytes. On the other hand, increased values for lymphocytes in addition to decreased values for neutrophils were observed only in the ACT-TR group (Figure 1). These results suggest the existence of a systemic activation of various hematopoietic lineages, antigen-induced secretion of parasite eggs [35–37]. Smithers et al. [38] demonstrated in experimental models that the cellular infiltrate observed in hepatic granulomas is predominantly formed by eosinophils and monocytes. Different studies showed that acute schistosomiasis patients have increased levels of circulating eosinophils [26, 32, 39]. Previous studies have also demonstrated that eosinophilia occurs between the 5th and 7th weeks after exposure to the parasite and may be induced by Th2 cytokines, such as IL-4, IL-10, IL-13, IL-9, and especially IL-5 [40]. Increased count of circulating monocytes was also observed by different studies [26, 41]. Moreover, Borojevic et al. [41] showed a delayed differentiation of bone marrow neutrophil granulocytes and blood monocytosis of *S. mansoni*-infected animals. These changes are compatible with modifications in the differentiation of bone marrow myeloid precursors, favoring the production of a monomacrophage cell lineage. Indeed, some previous studies show that during acute *Schistosomiasis mansoni* infection a higher percentage of neutrophil apoptosis is found, compared to patients with chronic illness or the control subjects [42]. Also, after the praziquantel treatment, we have a higher exposure of cryptic antigens which could be contributing to increase lymphocyte rather than neutrophil counts. Another important point to be addressed is that, after treatment, we have lesser levels of inflammatory cytokines such as IL-1-beta and TNF-*α*, important inducers of neutrophil mobilization to the peripheral blood during the acute phase of the disease.

Although our results do not show increased values for lymphocytes in the ACT group, in contrast with data obtained by Costa-Silva [32], a special interest in this study was the analysis of cytokines producing T lymphocytes, considering the importance of the specific immune response to solve the infection. Analyses of proinflammatory cytokines (IFN-*γ* and TNF-*α*) and regulatory cytokines (IL-4, IL-5, and IL-10) demonstrated higher percentage of TNF-*α* ^+^ T CD4^+^ lymphocytes in the ACT group after SEA stimulation in comparison with the control culture. Furthermore, in the control cultures, the results showed increased percentage of IL-4^+^T CD4^+^ lymphocytes in both ACT and ACT-TR group in comparison with NI (Figure 3).

The Th1-Th2 model of CD4^+^ T-cell differentiation is a well-established paradigm for understanding the basis of protective versus pathogenic immune responses for a host of important human pathogens [43]. Th1 cells secrete IFN-*γ* and IL-2 and promote cell-mediated immunity, while Th2 cells secrete IL-4, IL-5, and IL-10, among others. In this context, an important concept that evolved from the study of Th1 and Th2 cell responses is the fact that both responses cross regulate each other. Thus, IFN-*γ* downregulates Th2 cell development, while IL-4 and IL-10 antagonize Th1 cell differentiation [44]. In humans with acute schistosomiasis, higher levels of IFN-*γ* and lower levels of IL-5 are found in supernatants from PBMC compared to patients with chronic disease [19]. However, the results by de Jesus et al. [9] showed only high production of the proinflammatory cytokines IL-1, IL-6, and TNF-*α* in cultures of unstimulated PBMC from patients with acute schistosomiasis. Thus, our results suggest a mixed cytokine profile produced by CD4^+^ T cells. This cytokine milieu might be preventing tissue damage. In addition, development of a severe inflammatory disease during acute schistosomiasis has been documented in knockout mice for IL-4 and IL-10 genes [45, 46]. Interestingly, our results demonstrated predominant increased percentage of IFN-*γ* ^+^ and IL-4^+^ T CD8^+^ lymphocytes, following short-term incubation of whole blood with SEA in both ACT and ACT-TR groups and increase of IL-10^+^ cells only in the ACT group (Figure 4). This study is the first one to point out the relevance of CD8^+^ T lymphocytes in the immune response of acute phase of schistosomiasis. CD8^+^ T cells have been implicated in several immunopathological events during helminthic infection including schistosomiasis [47]. The presence of CD8^+^ T cells was observed in both early and chronic murine granulomas with an increased ratio of CD8^+^ cells during the chronic phase of the disease [48]. Studies developed by Pancré et al. [49] in murine model demonstrated that SEA was able to stimulate IFN-*γ* or IL-2 producing CD8^+^ T cells, suggesting a type 1 response induced by SEA. In humans, Oliveira-Prado et al. [50] showed a smaller percent of CD4^+^ and a higher percent of CD8^+^ cells in peripheral blood from patients with chronic schistosomiasis. Moreover, our group has previously described the putative role of CD8^+^ T subsets in controlling morbidity during human schistosomiasis, which was evidenced by the increased levels of activated HLA-DR^+^ CD8^+^ T lymphocytes in patients presenting intestinal clinical form (INT) of the disease and low levels of CD28^+^ CD8^+^ cells in hepatosplenic patients [51–53]. In addition, Teixeira-Carvalho et al. [54] observed increased percentage of IL-4^+^, IL-5^+^, and IL-10^+^ CD8^+^ lymphocytes following short-term SEA stimulation of whole blood samples in vitro in the INT group. Although the recruitment of CD8^+^ T cells by exogenous antigens (such as those derived from *S. mansoni*) primarily seems to be unexpected, recent studies have demonstrated that CD8^+^ T cells are prone to respond to extracellular antigens in infectious diseases [55, 56] via bystander activation triggered by persistent antigenic stimulation, cytokines milieu [52, 57, 58], or interaction with antigen-presenting cells (APC) that acquire exogenous antigens by phagocytosis and present them throughout MHC class I molecules [59–62]. Similarly, data of Montenegro et al. [19] showed that PBMC from acute schistosomiasis patients responded to SEA and SWAP by producing significantly higher amounts of IFN-*γ* and IL-10. A study carried out by de Jesus et al. [9] demonstrated the occurrence of higher levels of IFN-*γ* production in patients with acute schistosomiasis than those who have chronic infection. However, fewer patients with acute disease produced IL-10 in response to SEA. These authors also showed a positive correlation between IL-10 levels in SEA-stimulated PBMC and time after water exposure. On the other hand, Falcão et al. [63] showed that hepatosplenic schistosomiasis patients produced high levels of IFN-*γ* and low levels of IL-10 as compared to INT, associating this latter cytokine with the establishment/maintenance of the severe clinical forms. Interestingly, studies by Pedras-Vasconcelos and Pearce [64] demonstrated that mice infected with schistosomes present a regulatory pathway in which type 1 CD8^+^ cells, under the control of IL-4, dampen immunopathologic type 2 responses. Corrêa-Oliveira et al. [13] showed that the blockage of IL-4 and IL-5 using anti-IL-4 and anti-IL-5 antibodies significantly reduced the PBMC proliferative response to SEA antigens in acute, chronic intestinal, and hepatosplenic patients.

Another interesting data found was the high IL-10 producing cells in response to SEA in uninfected individuals. Like SEA is a complex mix of proteins, glycoproteins, and polysaccharides, this composition inducing could be inducing cross-reactivity sharing some epitopes with other antigens that previously sensitized the uninfected individuals.

Studies show that praziquantel is an effective drug in controlling infection with *S. mansoni*, promoting a reduction in egg excretion and regression of fibrosis in mice and humans [65–70]. We observed that one month after specific chemotherapy with praziquantel, the patients in this study showed similar changes in the number of eosinophils and monocytes, but had a specific decrease in the number of both neutrophils and lymphocytes as compared with NI and ACT or only NI, respectively (Figure 1). Some authors have demonstrated that chemotherapy-induced immune responses are heterogeneous, depending on the type of antigen used and time of analysis after treatment [70, 71]. In this context, we observed a higher number of IFN-*γ* ^+^ and IL-4^+^ T CD8^+^ lymphocytes in response to SEA in the ACT-TR group (Figure 4). De Souza et al. [72] demonstrated—six months after specific treatment with oxamniquine—that the IFN-*γ* levels increased significantly in response to SEA. Similarly, it was reported that patients with chronic disease living in endemic areas showed increased levels of IFN-*γ* after treatment in response to parasite antigens [73]. Probably, the increase in T-cell reactivity after chemotherapy can be explained by exposure of released antigens to the immune system following the destruction of worms by chemotherapy [43, 73–76]. In contrast to our study, De Souza et al. [72] have not found increased levels of IL-4 in patients with acute schistosomiasis after treatment with oxamniquine.

In conclusion, the data showed that a mixed cytokine profile is present during the acute phase of *Schistosomiasis mansoni*. One month after treatment with praziquantel there is a increase in the production of IL-4 and IL-10 cytokines following SEA stimulation in circulating lymphocytes from infected patients. This finding is associated with a reduction in their morbidity.

## Figures and Tables

**Table 1:** Ultrasonographic features of the study group.

Site of measurement	Reference values	CT (*N* = 12)	ACT (*N* = 21)
Longitudinal left lobe of liver	107.0	102.2 ± 16.0 (91.9–112.4)	99.7 ± 20.9 (89.9–109.6)
Anteroposterior left lobe of liver	70.0	42.7 ± 4.4 (39.6–45.9)	53.7 ± 7.6* (50.2–57.3)
Longitudinal right lobe of liver	150.0	134.3 ± 10.5 (127.3–141.4)	147.4 ± 11.5* (142.0–152.8)
Anteroposterior right lobe of liver	100.0	70.3 ± 10.6 (63.2–77.5)	84.3 ± 9.6* (79.7–89.0)
Longitudinal spleen	120.0	88.1 ± 7.0 (83.4–92.9)	116.9 ± 15.6* (109.2–124.7)
Anteroposterior spleen	70.0	33.7 ± 3.9 (31.0–36.3)	46.6 ± 8.5* (42.6–50.6)
Portal vein	<12.0	9.9 ± 1.2 (9.2–10.7)	10.5 ± 1.30 (9.9–11.1)
Hilar portal vein wall	<3.0	1.5 ± 0.3 (1.3–1.7)	2.0 ± 0.1* (1.9–2.1)
Splenic vein	<9.0	6.4 ± 1.3 (5.5–7.2)	6.5 ± 0.9 (6.1–6.9)
Superior mesenteric vein	<9.0	6.4 ± 0.8 (5.9–7.1)	6.2 ± 0.8 (5.8–6.6)

Values are represented in mm as mean ± standard deviation and 95% confidence intervals.

*Represents a statistically significant difference (*P* < 0.05) among groups CT and ACT.
